# "The Hospital" Nursing Mirror

**Published:** 1900-05-19

**Authors:** 


### "The Hospital" Nursing Mirror.

Being the Nursing Section of "The Hospital."

[Contributions for this Section of "The Hospital" should be addressed to the Editor, The Hospital, 28 & 29, Southampton Street, Strand, London, W.C., and should have the word "Nursing" plainly written in left-hand top corner of the envelope.]

### Notes on News from the Nursing World.

### MORE NURSES FOR THE FRONT.

That the War Office still consider the number of nurses at the front inadequate is shown by the fact that on Wednesday the following members of the Army Nursing Service Reserve embarked for South Africa: Misses E. E. Adkins, A. B. Briscoe, E. M. Dasent, D. Fletcher, C. E. Gregory, H. Wohlford-Hansen, F. Low, A. J. Moffat, F. A. Rhodes, and H. M. Young. Miss Helena Mary Young was trained at the Hospital for Women, Soho, and has been staff nurse at the Metropolitan Hospital. She holds Mrs. Creighton Hale's massage certificate. Since 1898 she has been a private nurse in connection with St. John's House, Norfolk Street.

### AMATEURS AND ARMY NURSES.

A correspondent, observing our statement that unless or until an individual instance of the appointment of an amateur as nurse in a military hospital at the front can be proved, the statements about amateur nurses occupying official positions do not deserve credit, writes: "A superintendent sister of the army states that when Sister —— got up to Maritzburg she found a lady amateur, who had once been 'guinea pig' for three months in a London hospital, put in charge of 50 soldiers in one of the camp wards. Another, who had had no training at all, was also a charge nurse, and she could not read one of the symbols used for the medicines." "Both these ladies," adds our correspondent, "were in receipt of two guineas a week and allowances from the Government." It is quite possible that, pending the arrival of nurses of the Army Service, the authorities were glad to make use of the best help obtainable on the spot, but this is a very different matter from the War Office employing and paying amateurs in preference to trained nurses. Lord Wantage, in a letter to the *Times*, admits that in addition to the Army Service and the Army Service Reserve, there are nurses employed "locally" in South Africa, "for whom the Central British Red Cross Committee are in no way responsible," but it is not clear that he alludes to amateurs.

### MORE "NURSING" ANNOUNCEMENTS.

It is stated in the *Daily Chronicle* that Lady Sykes, who went to the front "in the smartest of nursing costumes," has now returned to England, and is about to publish a volume "describing how she nursed the wounded at Estcourt." We reserve comment on the "nursing" done by Lady Sykes until we have seen the account of her experiences. Another lady of title—Lady Briggs—writing to the *Morning Post* from South Africa about the Simonstown hospitals, alludes to "Miss Mary Kingsley and other nurses," although before her departure Miss Kingsley took the special precaution to signify that she had no claim to be regarded as a nurse.

### THE NURSING QUESTION IN THE FAR EAST.

If the authorities of the Jubilee Hospital at Peking had asked the Colonial Nursing Association to supply them with nurses, it is unlikely that they would have experienced the difficulty which, according to a report in the *Peking Times*, has been harassing the committee. It appears therefrom that in April last two nurses arrived in the Chinese capital from England, but that one of them did not take up her appointment, "not being duly qualified." The other nurse had, therefore, to assume responsibility for the care of all the patients, and at one time the position seemed most critical, for the only nurse herself fell ill, and was quite unable to attend to her duties. Fortunately, her illness was of short duration, "but," as our contemporary says, "the absolute necessity for further assistance was in this way vividly illustrated." There can be no room for doubt upon this point, but we cannot understand how it was that Dr. Cantlie, who, according to the report, undertook to secure and send out the nurses, engaged one who was not duly qualified. If people are willing to pay a reasonable salary, and treat their nurses well, there are plenty of good trained nurses quite prepared to go out to the Far East or anywhere else.

### A BISHOP ON NURSES IN WORKHOUSE INFIRMARIES.

On Monday the Bishop of Winchester, whose wife performed the ceremony of opening a new infirmary at the Farnham Workhouse, in the course of an address said it was something to be thankful for that the word "workhouse" was now a misnomer, and "that they would not now find that the nurses in the workhouse infirmaries were such as those depicted by Charles Dickens." We are afraid that Dr. Randall Davidson takes a little too optimistic view of the situation. There have, of course, been immense improvements in the provision made for the wants of the destitute, and in the *status* and *personnel* of the nurses. But a good deal remains to be done in some parts of the country; and, though the nurses such as those depicted by Dickens may be almost extinct, the standard of nursing in many infirmaries still requires to be raised.

### UNTRAINED NURSES IN WORKHOUSE INFIRMARIES.

At a meeting of the Mutford and Lothingland Board of Guardians, Dr. Bowling made the extraordinary statement that about twenty nurses employed by the Board had only stayed "a month or two." The subject under discussion was, of course, the usual difficulty of obtaining nurses, upon which some light was thrown by the answers of a nurse who was summoned before the Board to explain a letter she had written to a local paper asserting that the reason she applied for her post was that "she wished to test for herself why the workhouse had such a bad name for frequently changing nurses." In reply to Dr. Bowling's questions, she said that she had been a nurse in a convent for three months, and had been nurse-attendant at Bournemouth. But it ranspired that she had only "thought of" going into "thorough training" before she entered the infirmary. Without touching upon the particular dispute between the nurse and the Guardians, we may observe that so long as the latter are content to engage persons to nurse in the infirmary who, admittedly, have not been fully trained, they must not expect to receive applications for vacancies from members of the profession who hold certificates of competence.

### THE HONG KONG MEMORIAL TO PLAGUE NURSES.

In addition to the window which has been put in St. John's Cathedral, Hong Kong, in memory of Elizabeth Frances Higgin and Emma Gertrude Ireland, who died of plague contracted while on duty at the Government Civil Hospital, the Memorial Committee have been able with the surplus of the fund subscribed, and after consultation with the matron, to purchase a handsome clock and a large case of plate and cutlery for use in the Sisters' quarters at the hospital. Miss Barker writes to the committee that this memorial is very highly appreciated by the Sisters, both in memory of their deceased fellow workers and also of the kindness of the community. A small balance still remaining was spent in adding a few books to the Sisters' library. The subscriptions to the fund amounted to 2,546 dols.

### THE ALTERATIONS AT THE LONDON HOSPITAL.

When the extensive alterations and improvements in progress at the London Hospital are completed, the Jewish wards above the obstetric wards will be arranged for the reception of maternity cases, and a definite course of instruction will be offered to pupils in monthly nursing and midwifery. The number of beds to be devoted to this branch of the work is not yet definitely settled, but it will be between 20 and 30. The nursing at the isolation cottage, now being built, will be managed by the permanent staff, and will prove useful to the nurses, as it will afford them an insight into the special methods of treating many infectious complaints which are not suitable for a general hospital. It is not intended to keep fever cases in the isolation hospital; they will be sent, as hitherto, to the metropolitan fever hospitals.

### THE NURSES' HOTEL.

Having outgrown its present premises, the Nurses' Hotel in St. Andrew's Chambers, Wells Street, will be moved in June to a building now approaching completion in Mortimer Street, W. Every arrangement is being made which can contribute to the comfort and convenience of the inmates. There will likewise be a club in connection with the hotel, which is also open to non-residents. The rules, which are carefully drawn up, have been submitted to us. Full particulars can be obtained on application to the secretary, 14, St. Andrew's Chambers, Wells Street, W.

### THE CEYLON NURSES' ASSOCIATION.

The colony of Ceylon has the same difficulty as some of our provincial towns in obtaining support from the public for its nursing association. At the annual meeting the curious statement was made that the number of subscribers on the roll in 1899 was over 400, but only 250 of these had subscribed. This means either that their names were retained on the list without their permission, or that someone had failed to collect the amounts due. A more unsatisfactory feature still is that in the list of liabilities is a large amount for salaries due to nurses. Salaries should, of course, be promptly paid, and unless such obligations can be discharged without difficulty, they should not be incurred. It may be hoped, however, that, as the Planters' Association have taken the nurses' association under its ægis, the financial position will be greatly improved; and it is very satisfactory to learn that Miss Ramsay, who was recently sent out by the Colonial Nursing Association, is doing excellent work in the dual capacity of lady superintendent and secretary.

### NURSING CONSUMPTIVES AT VICTORIA PARK HOSPITAL.

The progress of the Victoria Park Hospital for Consumption and Diseases of the Chest during the last two and a-half years has been rapid and admirable. At the beginning of that period only 16 of the 164 beds were in use, now 126 are open. Two new balconies have been added; they are very wide, extend round the south-west corner of the building, and are well protected from rain. On these balconies the patients spend nearly the whole day. They recline at their ease on basket lounges, supplied with cushions, rugs, and hot-water bottles if it is at all cold. The nurses say that the treatment is most popular, and that so far, the results have been very satisfactory; they think, too, that it is very probable the younger patients will continue the regimen after leaving the hospital, but that it is very doubtful whether the older people will take the trouble. Of course, the nursing staff has been largely augmented to cope with the extra work caused by the reopening of so many beds. The matron, Miss Beatrice Jones, has been granted six months' leave of absence for work in the front. She is at Bloemfontein just now, but her place is filled by an old fellow probationer at "Bart's," who has been her assistant for some time. At present there are 25 nurses, and a night sister and two day sisters on each floor. The matron gives two years' training, and has arranged that an additional year's training at a general hospital of 40 beds, either before or after the probationer's sojourn at this hospital, shall qualify the nurse for a three years' certificate. The Royal British Nurses' Association has consented to register nurses thus qualified. It is the aim of the authorities to make the nurses' surroundings as bright and attractive as possible, and this year a tennis lawn has been laid out for their amusement. As it is situated in front of the hospital there is a good view of it from the balconies, and doubtless the patients will often derive pleasure in watching the games. It is satisfactory to learn that there has been no case of consumption among the nurses.

### A NURSE OF NINETEEN.

Nineteen is an early age for a young lady to take up nursing, but the Belfast Board of Guardians cannot be congratulated on the manner in which they have treated Miss Curry. Having engaged her as a temporary nurse, with a full knowledge of her age, they refused, entirely on account of her youth, to appoint her to a permanent post in the infirmary. The proper course would obviously have been to have observed the limit prescribed by the Irish Local Government Board, though the latter are somewhat to blame, because it appears that on a previous occasion they passed a young lady whose age was open to a similar objection. The incident shows the importance of adhering to regulations, which, if not unexceptionable, should be maintained and obeyed as long as they are in operation, and also that young people seeking to enter upon nursing work should make enquiries as to the future before tying themselves as to the present.

### BARRY NURSING ASSOCIATION.

The annual report of the Barry District Nursing Association and Accident Hospital confirms the statement that nursing in the thriving port of Barry is highly appreciated by the working classes. During last year 1,050 cases were visited and 16,927 visits paid, while the financial position is indicated by the fact that the receipts were £1,463, as against expenditure £1,373. Another proof of the popularity of the association is that, under the auspices of the Barry Trades and Labour Council, a collection on its behalf was made at a recent labour demonstration. Miss Blanche Sykes, having been appointed to the charge of the Accident Hospital, lately taken over by the District Council, Miss Aldis, of Stockport, who succeeds her, has entered upon her duties as superintendent of the association.

### A NURSING INSTITUTE FOR HINCKLEY.

A large bazaar was recently held at Hinckley to augment the fund for the Jubilee Cottage Hospital and Nursing "Institute," now being built in Mount Road, to commemorate the sixtieth year of her Majesty's reign. The present hospital having been found quite inadequate to meet the requirements of the growing population of the town and neighbourhood, and being, moreover, only of a temporary nature, a site for a permanent building was acquired some time back, and the sum of £1,500 collected towards the new cottage hospital, which, when furnished, will cost about £3,000. At the bazaar, the town, with the neighbouring villages of Barwell, Burbage, Aston Flamville, and Earl Shilton, worked so well that they realised over £1,200 in two days, so that, with but a small addition, the new hospital will be enabled to start free of debt, and, through the instrumentality of Dr. Pritchard, as many as eleven nomination beds have already been promised.

### SUTHERLAND BENEFIT NURSING ASSOCIATION.

At the half-yearly meeting of the Sutherland Benefit Nursing Association held in Golspie, Mrs. Taylor, Dornoch, presiding, the treasurer, Mr. James Morrison, submitted some particulars as to life assurance for the nurses, but as there was a small attendance of members, it was postponed for further consideration. The Secretary read a letter from Dr. Sutherland, formerly M.O.H. for the county, in the course of which he said: "As I am at an early date leaving this county I beg hereby to intimate through you my resignation as a member of the Executive Committee of the Sutherland Benefit Nursing Association. I do so with the sincerest regret, for it has been to me from the very beginning the greatest pleasure to work with the association. I always perceived that the welfare of the people, and the amelioration of their condition was the main object kept in view; and I am sure that the product of the association's unselfish labours will be still more apparent in the days to come." The committee resolved to record their regret that Dr. Sutherland's official connection is severed, and their appreciation of, and thanks for, his interest and help.

### "A QUEEN'S NURSE."

Daddy, says a correspondent, was eighty-seven, and had never been to school, neither had he more than once been fifty miles from his native village. Of course he knew there were doctors for folks as "needed 'em," but he had never needed one till now; so the first visit of the district nurse was an event in Daddy's old age. The nurse did everything that was necessary without any comment from Daddy until she began to write her notes, when he burst forth with enthusiastic admiration. "Eh! but it's a gran' thing to be a scholard!" By the next visit Daddy had become more confident, and began to elicit a little more information for the benefit of his next visitor. "Na doot ye has a husband? Nay? Ah, well! ye lives in a gran' house, and has good things to eat! Ye hanna a house o' your own? Why, I thowt as ye come from the Queen ye would have a fine, big house; but mebbe your rooms are mighty grand."

### SHE WAS FOND O' PEPPERMINTS

A patient's partiality for a nurse is not always a source of gratification. "There is an old lady in one of my wards," writes a correspondent, "who shows her fondness for me by occasionally giving me what she calls suckers (meaning sweets). To avoid hurting the poor old thing's feelings I always take them and pass them on, if they are not too dirty, to a child in another ward. Some time ago I was going through the ward with our visiting committee. As we went along my grannie followed, and called loudly, 'Wait a minute, ma'am.' Of course, the gentlemen turned to see what was happening, and, in a silence which was profound, the old dame, who would not be put off, fumbled in her pocket for a moment, and finally produced two very dirty sweets and a piece of butterscotch. She put back the butterscotch, and, with a great deal of ostentatious display, presented me with the sweets, remarking to the company as she did so, 'She's mighty fond o' peppermints, is our nurse.' The visitors roared with laughter, in which I was constrained to join, though my feelings at the incident may be better imagined than described."

### SHORT ITEMS.

The nurses of the Metropolitan hospitals and of the London nursing institutions are again invited to spend a "day of rest" at Hertingfordbury on Wednesday, June 27th. The addresses at the two short services held in the parish church will be given by the Dean of Windsor. As hospitality is completely provided, the expense to the nurses is limited to the railway fare of 2s. 6d. Any who may wish to be present should communicate with the Rev. A. G. Locke, St. George's Hospital, Hyde Park, or with Canon Burnside, Rector of Hertingfordbury, Hertford.—Last year, for the benefit of the Hampton Nurses' Fund, a comic cricket match was arranged, and yielded between £30 and £40. It has been arranged to have another match on the same lines this season.—At the last meeting of the Committee of the Middlesbrough Nursing Association, a reference was made to the kindness of the Imperial Tramway Company, who give the district nurses free passes over their system.

### Lectures on Medicine to Nurses.

By H. A. LATIMER, M.D. (Dunelm), M.R.C.S. Eng., L.S.A.Lond.; Consulting Surgeon, Swansea Hospital; Past President of the South Wales and Monmouthshire Branch of Brit. Med. Assoc.; Past President of the Swansea Medical Society; Hon. Life Member of the St. John Ambulance Association, &c.

### THE ELIMINATIVE TREATMENT—GERM THEORY—GENERAL PRINCIPLES OF TREATMENT.

I THINK you must see from what I have said about the Antiphlogistic, Humoral, and Eliminative theories that there was good in all of them; the fault was in the predominance which each one obtained at the time of its acceptation, and in the exclusion of all others at the time in which it was put into force. Rightly considered, and used with due moderation, it was a wise thing when fever was prevailing, and when the digestive organs were out of gear, to give a diet which did not call for the full activities of the stomach and eliminative organs to dispose of the same. The wrong application of the theory was the withholding of nutritious foods and the lowering measures which were adopted for stopping disease which, in many cases, was not capable of being arrested by any such means. You will readily perceive that this and the other systems I have spoken of were faulty in their very inception, inasmuch as they ignored the causes and true nature of disease, and aimed at treatment of symptoms, which are but the cry of suffering organs—they did not grapple with the essentials of maladies. Moreover, the measures advocated during the time they held sway were nearly always of a lowering character, and subsequent experience has taught us that the animal strength requires careful conservation during disease, and that measures which tend to lower and reduce it at that time do an infinite amount of harm.

If any one idea regarding the causes of disease can be said to prevail strongly over others at the present time, it is that known as the Germ or Microbe theory. A great number of diseases are now known to be the result of an infection of the blood by bacteria — this being the generic name for a great variety of minute organisms which convey infection, either by their mere presence or by the poisons which they secrete. These bacteria are of minute size and of various shapes, the three main forms being rods, or bacilli, dots, or cocci, and corkscrew-like bodies called spirilli. Some of them are endowed with powers of movement, whilst others are quiescent. They all possess distinctive forms by which they can be distinguished from each other under the microscope, and after especial stainings by chemical reagents; and in many cases the efforts which they produce are now recognised with certainty. Many of them gain access to our bodies by being carried into the lungs with the air we breathe, for they float about in the atmosphere and form a constituent part of the dust which is all around us. Some are introduced through foods and drinks. They all have specific powers of doing mischief, and if they can suitably locate themselves, breed diseases, each after their kind. They may be likened to seeds which, when sown in fertile soil, redevelop the plants of which they were the offspring. They breed truly. Just as the seed of a daisy will develop into a daisy plant if it falls on suitable soil, so the micro-organisms of tubercule or of diphtheria will, under suitable circumstances, produce attacks of tubercular phthisis or diphtheria. Variations in the types of these and other germ-induced diseases are due to the circumstances, habits, constitutions, and environments of the people who have been attacked by them, and also to the circumstance that two or more germs may be present in an illness, and may be producing their specific efforts at the same time. Thus, for instance, putrefaction is the peculiar result of the toxins or chemical products of certain germs. The toxins of the dot-like micro-organisms called staphylococci and streptococci will set up suppuration and abscesses if allowed to develop in the blood and tissues, and if these should be operating at the same time as the tubercle or diphtheria microbe, the course of either of these diseases will be complicated by suppurative processes. When I come to speak of diseases which are caused by microbes, I will deal appropriately with their bacteriological aspect; what I have now said on the subject will, I think, suffice for the present.

Apart from the special circumstances of each individual disease, the general principle dominating the treatment of maladies, at this time, is that of giving rest to impaired organs, so that the natural tendencies to recovery which exist may come into play. It is a commonsense view of things to believe that if an organ is out of working order its chances of recovery will be increased by its being rested as much as possible, until such time as it can again resume its full functional activity. But how is such repose to be secured in the case of important viscera? How can the heart, whose function it is to act as a driving machine of the blood, be rested from its labours? and how, as another example, can the kidneys be lightened in their burden of separating from the blood the various salts and excrementitious matters which must be removed therefrom if health—nay, even life itself—is to be maintained? If a limb is injured we know that it can be secured in splints or bandages, and so, as it were, put aside until the injury has been repaired; but I can picture to myself the novice inquiring how can the vital internal organs be given repose? The answer is furnished by an application of the knowledge we possess of the laws of physiology. We know, for instance, that the heart beats with increased force and rapidity if it has to overcome any obstacle to the circulation of the blood, either in the general current of the circulation or within the organ itself. The circulation of the blood, also, is like the circulation of any other fluid, and a driving force will have to be increased in strength if it has to circulate it uphill. Thus, if you count the pulse of a person lying down, then sitting up, and then standing, you will find that with each change of posture its frequency is increased. This shows that the heart has had to beat oftener than it did before, as the column of blood it had to keep moving offered increased resistance to its pressure. You will see, then, that one plan for lessening the heart's work consists in adopting measures which facilitate the passage of blood through its vessels. In conditions of debility the heart suffers from a reduction of strength as well as other parts of the body, and rest is given it by any foods or drugs which give it more vigour. Thus the drug Digitalis (Foxglove), which has, among other properties, a sedative action on the heart, produces such a tonic effect that each contraction of the expelling chambers is made to be thorough. The result follows that the organ does its work with fewer contractions than it made in its weakened state, and has more time given it to pause between them, so that an opportunity is given it for rest. By the nature of its office it can never stop beating whilst life exists, but it can be stimulated to work in so rhythmical and efficient a manner that it can pause between the beats and so secure its full measure of physiological repose. I will not for the present go further into this question, nor need I dilate on the subject of rest to the kidneys which is afforded by diverting some of the matters which they carry out of the system to the routes furnished by the skin or bowels. You will readily understand that if, during kidney disorders, food which, when metamorphosed, has to be removed from the system by these organs is withheld, they will have a lessened amount of work to do in consequence, and so will be given a chance of rest which induces to recovery from disease.

### Miss Florence Nightingale.

PRESENTATION BY THE NIGHTINGALE NURSES.

Saturday, and not Sunday, nor Monday, nor Tuesday—as various daily papers in their wisdom have intimated—was the eightieth birthday of Miss Florence Nightingale, and, as our readers are aware, the Nightingale nurses had arranged to celebrate the event in a special manner. It was desired to include as many as possible of the names of those who had received their training in the Nightingale Schools at St. Thomas's Hospital and at St. Marylebone Infirmary amongst the signatories of the congratulatory address, and the task of tracing their whereabouts and of writing to so large a number has been no light undertaking. But the result has been eminently successful. As many as 650 signatures were obtained from all parts of the world, Australia, various parts of India, South and West Africa, Egypt, Palestine, Algiers, China, North and South America, Russia, Finland, Denmark, and other Continental countries, besides Great Britain and Ireland.

### The Oldest Nurses.

It has always been believed that Mrs. Dacre Craven (F. Lees)—whose name must be familiar to all nurses as the organiser of the National Metropolitan Nursing Work in London, now merged in the Queen Victoria's Jubilee Institute for Nurses—was the oldest Nightingale nurse living, not in point of age, but of training. But it appears that there still exist three nurses who entered the school before Mrs. Craven, all of whom signed the address—Mrs. Caroline Stone, who entered in 1860, a month after the institution was opened; Sarah Anne Terrot, of Edinburgh; and F. Lovesay.

### How the Names were Collected.

The first idea in collecting the names had been to forward the sheets to each nurse and ask her to sign, but as soon as it became apparent how scattered was the field of their labours, the insuperable difficulties of doing so caused the original notion to be abandoned, so that the names, as they were received, were simply neatly entered by one person into the handsome brown leather album lined with white silk which had been selected for their reception. The nurses were grouped together according to the years in which they had entered the school. Two in 1860, one in 1862, one in 1866, one in 1868, two in 1869, and one in 1870. After that the numbers each year rapidly increased.

### The Address.

Upon the first page was the illuminated address, which ran as follows:—

"Dear Miss Nightingale,—We, as your nurses, speaking for ourselves and many others who have been trained in your schools and are proud to bear the name of our dear chief, trust that you will allow us to offer you our hearty congratulations on this your eightieth birthday. We rejoice that you are still able to see some of us from time to time, and to take a lively personal interest in our work. We feel grateful to you, not only for the benefits which you have conferred upon us individually, but also for having, by your example and wise direction, created a noble calling for women generally. We beg you will accept this book, containing our names, and a basket of flowers, as our birthday offering."

Then followed the signatures, and at the end of the book were added eight of the most recent photographs of St. Thomas's Hospital.

### No Formal Presentation.

There was no formal presentation, as Miss Nightingale objects to receive deputations, but the flowers—a very large basket of choice flowers tied with pink ribbons, with a tiny knot of red, white, and blue introduced for the sake of patriotism—and the book were conveyed to Miss Nightingale's house on the afternoon of Saturday. Though too tired with the excitement of the day to receive the representatives who had taken the gift to her house, she expressed the hope that another day she should be able to see the bearers and have a chat with them.

### The Howard de Walden Nurses' Home and Club.

(The Nurses' Co-operation.)

On Wednesday the newly-built Nurses' Home and Club in connection with the Nurses' Co-operation (8, New Cavendish Street) was opened to inspection by invitation, and next week will see the nurses fairly in possession of their very comfortable quarters. A home and club have long been wished for by the committee of the Co-operation for the exclusive use of their large staff, now numbering five hundred nurses, and the nurses themselves have found the "housing question" a real difficulty. The late Lady Howard de Walden, warmly interested as she was in the Co-operation, and learning from Miss K. Phillipa Hicks—so long its superintendent—that the surplus fund gradually accumulating from the small percentage for "office expenses" paid by the nurses was intended to meet the cost of a "Home" in the future, generously offered to provide half the cost of a suitable building if the plans suggested and the site selected by her were acceptable to the committee.

### The Exterior.

The new building is, perhaps, best described as a residential club. It is solely for the use of Co-operation nurses, who by payment of a yearly subscription are entitled to its advantages, either as residents or as members of the club. They can have bedrooms for as long or short a time as they desire, paying by the night or week, and the new home provides private hanging cupboard and boxroom, while meals being arranged for on the "restaurant" principle, their convenience is catered for in every possible way.

It must be confessed, from an architectural point of view, that the exterior of the Home is the reverse of pleasing, though from the aspect of practical cleanliness it has undoubted advantages. The entire front is faced with white glazed bricks, "having a simple flat ornament or relief in black glazed bricks with cornices, &c., in Doulton's glazed ware," this method of treatment being in accordance with Lady Howard's suggestions, and the cost thereof being defrayed by her.

### The Interior.

Inside the entrance is very pleasing. Terrazzo is used for the flooring here and in the kitchens, and everywhere spotless tiles, relieved with pale greens and yellows, give a delightful sense of cleanliness. On the ground floor are the office, cheerfully papered with yellow, a quite charming sitting-room for the residents, and another for club members only, Miss Wells' (the matron) private sitting-room, cloakroom, lavatories and bath-room, and the dining hall and restaurant, with servery attached. The dining hall is a most pleasant place, yellow-tiled and well-lighted, and fitted with small tables. Here the china calls for admiration, plain white, with the Co-operation badge in dull red. The sitting-rooms are made especially bright by the loan of a number of good pictures from Mr. Charles Lamport, and also some beautiful statuary, and a gift of pottery from Messrs. Doulton. The white-tiled kitchens, light and airy, with every modern convenience, and ample larders must delight the heart of any housekeeper. The upper floors contain 43 bedrooms, cosy little rooms, with just enough of the right sort of furniture, and unlimited cupboards. The building is heated throughout with hot-water, a good many bedrooms also having fire-places. The walls of the bedrooms are distempered in pale tints, and the dainty linen bed-spreads match in tone. Linoleum is used everywhere, with rugs. There is a sanitary wing built out from the main building containing bath-rooms and w.c.'s, and a room, with two beds, is set apart for a sick-room. The lighting is electric throughout.

It might be mentioned that a library will be an early need, and that books towards this object will be very gladly received by Miss Wells.

### Our Duty to the Dying.

By a Nurse.

There is a difficulty which has presented itself to me again and again in my nursing life. It is the question whether it is right or wrong to tell the truth to a dying patient. Can it be wrong to tell it? Can it be right to conceal it? I often wonder if it is not in mistaken kindness that we fence about the facts of a case. Is it kind and fair to jump to the conclusion that our patient is a coward and unable to face the inevitable in a manly spirit, unwilling to accept the law of the universe, the will of the Creator in Whose hands he lies? Why should he be denied his chance to prepare for the great change, and to grow accustomed to the overwhelming thought, if that can be?

To know, and to be prepared are not, of course, one and the same thing. Many die suddenly, surely not altogether unprepared, and many more know what the end must be, yet this knowledge does not always constitute a ready state. Sometimes I am sure the patient knows as well as we do that he is dying, and it is disconcerting to think how often he must laugh in his sleeve at our elaborate deceptions. But often he has no idea, and then a great responsibility rests with us who know. How shall we meet it? How shall we answer for it?

Doctors, I believe, differ on this point, as upon many another. As a nurse I have always considered myself in duty bound to respect their views on the subject, and to let no look or word of mine betray to their patient that which they wish concealed. Professionally I believe this to be right; but morally, is it so? Must the clergyman be excluded lest his utterances should excite the patient and cause a rise of temperature, or lest the patient should guess that to be true which is true? I have seen less consideration for the patient's welfare displayed in clinical lectures. All clergymen are, unfortunately, not "sons of consolation"; but many are, and often and often have I known my patient to be "wearying" for the words which they alone can speak.

### A Personal Experience.

I think the temperament of the individual should perhaps be taken into consideration. I once nursed a patient who was informed before my coming of his approaching end. He was handed over to me, and I was told that he might live another day or two. That was in the beginning of May, and he lived until July. The knowledge he possessed was not conducive to a peaceful end. Harrowing farewell scenes had to be gone through with his mother whenever he felt a little worse. "Nurse" must not leave him for a moment, lest he should die in her absence. The fear of death hung over him all the time like a black shadow. If all temperaments were like his I should certainly be tempted to say, whether rightly or wrongly, "Don't tell."

### A Contrast.

But there are others who watch for the Angel of Death, and to whom it is no charity to prolong the time of watching. Of such are those whose pain they say:—

"Passeth not, nor will pass; and only this
Remains for us to look for—more of pain,
And doubt if we can bear it to the end."

A sufferer of my acquaintance, who has lived now for years lying on her back in a darkened room, said the other day: "I had a very trying time a few nights ago. I remembered a sermon I once heard in which the preacher said that when anyone dies that soul has to spend a thousand years in Hades. The idea took hold of my mind, and I lay and fretted about it all night. In the morning I was worse when the doctor came. But the next night I remembered something else. The words of Christ upon the cross came back to me and comforted me, 'To-day shalt thou be with Me in Paradise!' And I knew that these words were true and the others false; so I fell asleep and slept well. When the doctor came in the morning he said I looked brighter. I smiled, for I thought he little knew how much reason I had to be brighter."

Yes, indeed, a thousand years (that is, a long, indefinite period) interposed between us and rest. What consolation is there to be found in such a thought? It was as if Christ had said to the dying thief, "You shall not die, after all. I will release you before it comes to that, and your torn and lacerated body shall be let loose from your cross. You shall live to be an old man." What a mockery of recovery that would have been! I do not think that getting a little better, being able to "get about again," is always the change most to be desired. Rather than build up false hopes is it not kinder to our patients to foster in their minds a more cheerful view of death?

"O, . . . Heart, be comforted,
And, like a cheerful traveller, take the road,
Singing beside the hedge! What if the bread
Be bitter in thine inn, and thou unshod
To meet the flints? At least it may be said,
'Because the way is short, I thank Thee, God!'"

### Appointments.

Fountain Fever Hospital, Lower Tooting.—Miss Irene Webb has been appointed Assistant Matron. She was trained at the London Hospital, and has since been assistant nurse at the Western Fever Hospital; ward sister at St Saviour's Infirmary; head nurse at the Royal Hospital, Putney; charge nurse at the Western Fever Hospital; and night superintendent at the Grove Fever Hospital, Lower Tooting.

Nursing and Convalescent Home, Wootton-under-Edge.—Miss E. Higgins has been appointed Matron. She was trained and for nine years held the post of ward sister at the Royal Hants County Hospital, Winchester. She has been the recipient of numerous presents, including a table and an easy chair from the nursing staff.

### Minor Appointments.

Dewsbury General Infirmary.—Miss Lilly M. Park has been appointed Charge Nurse. She was trained at the Grimsby and District Hospital and District Infirmary, Ashton-under-Lyne, and has since been private nurse at Eastbourne Nursing Institution, and night superintendent at Hereford General Infirmary.

Chichester Infirmary.—Miss H. F. Letitia Bown has been appointed Night Sister. She was trained at St. Mary's Hospital, Manchester, and has since been ward sister in that institution.

### TRAVEL NOTES AND QUERIES.

Rules in Regard to Correspondence for this Section.—All questioners must use a pseudonym for publication, but the communication must also bear the writer's own name and address as well, which will be regarded as confidential. All such communications to be addressed "Travel Editor, 'Nursing Mirror,' 28, Southampton Street, Strand." No charge will be made for inserting and answering questions in the inquiry column, and all will be answered in rotation as space permits. If an answer by letter is required, a stamped and addressed envelope must be enclosed, together with 2s. 6d., which fee will be devoted to the objects of the "Hospital Convalescent Fund." Any inquiries reaching the office after Monday cannot be answered in "The Mirror" of the current week.

Belgian Seaside Resorts (St. Francesca).—Ostend is very expensive—nothing really reasonable. At Blankenberghe the Hotel du Phare will take you from 6 fr. a day. This is on the Digue. In the town, Etoile d'Or, more expensive, 8 to 10 fr. Grand Hotel Godderis, on the Digue, professes to take you from 7 fr. a day. I think I should myself go to the Hotel du Phare. There is a very cheap place called Knokke, close to Blankenberghe—Grand Hotel de Knokke, on the beach, from 5 fr.—but Knokke is very quiet, which might not suit you. It is, however, in easy communication with Ostend and Bruges, as well as Sluis and Middelburg, in Holland.

### The Association of Asylum Workers—Annual Meeting.

Sir James Crichton Browne, who presided at the annual meeting of the Association of Asylum Workers on Monday, said that it was hard on asylum nurses and attendants that they should be the only branch of the service to receive no pension on retirement. He liked, he continued, to think of them as soldiers, fighting through a long campaign, and cheerfully enduring many hardships, facing the combined forces of insanity. "Is the country to say," he inquired, in moving the adoption of the report and balance-sheet, "when you have gone through all this, and wish to retire, 'Go to, we have no further need of you?'" He thought not. He thought the time would come when it would be seen by the British public, and by those county councils that are at present obstructing legislation on the subject, that it was a question that could not be shelved.

### M.P.'s in a Soluble Condition.

Members of Parliament, Sir James went on, are in a soluble state at election time, and as a general election cannot be so very far off, the plan of action is to go on pegging away until they can be got to see the duty of the country towards those who perform so important a work for so large a proportion (comparatively speaking) of the population. And if England is to maintain her prestige on the score of the good condition of her public institutions, and not be left behind other countries, she will certainly have to wake up; the status of asylum nurses should be such as to attract the best women in the profession, and this can hardly be the case until the pension scheme is properly adjusted.

### Domestic Servants as Nurses.

In the course of his speech the Chairman gave a very interesting little historical sketch of lunatic asylums as he knew them. "When I had my first appointment in this country," he said, "I noticed that all the female attendants came into the dining-room of the medical superintendent to prayers every morning at nine o'clock, and I used to wonder how it was that the male attendants were excluded from this form of the means of grace. The fact was that the women nurses were in the position of domestic servants at that time and did not rank as nurses at all. Since then asylums have gradually been transformed from being prisons and Dotheboys Halls; they have become institutions, and I look forward to the time when they will be hospitals."

### Nursing the Insane as a Vocation.

As yet, though the class of nurses is improving, and many are now drawn from the educated classes, the life has not proved sufficiently attractive to the nursing profession as a whole. A great deal is being done to instruct the nurses in physiology, hygiene, &c., and this is just as it should be, for, to quote the Chairman again, a mental nurse should be a nurse and something more. She should be highly skilled in medical and surgical knowledge; she should be capable of going to South Africa to nurse the wounded, and then she might go on to India, where miserable provisions are made for the insane.

### New Openings in India.

Sir James hoped that when the Viceroy (Lord Curzon) was at liberty to attend to this important question of provision for the insane, a very great deal would be done to reform the state of affairs in India, and, he added, that when this was effected there would be great openings for nurses there.

### Other Speeches.

But the Chairman's speech, though it was the longest and full of interest, was not the only one of importance. Dr. Shuttleworth, the hon. secretary, had to reply to the eulogistic remarks made about him, in which it was said that, in accordance with his name, he was occupied, in speeding to and fro, weaving the Association of Asylum Workers into a goodly fabric. Dr. Sidney Copeland spoke of the impression made on him the first time he went into an asylum, and how, familiar as he was with methods of nursing the sick in body, it was a perfect revelation to him to see how thoroughly the sick in mind were cared for. He observed that, although the Association had only been in existence for three years, yet it could show a membership roll of nearly four thousand. He thought it proved that the spirit of co-operation was on the increase.

### A Good Word for the County Council.

Dr. Hayes Newington, treasurer of the Medico-Psychological Association, said a good word for the county councils. He believed that their desire was to do the work entrusted to them as well and as economically as possible. But the case was urgent, and the society of which he was treasurer, through its Parliamentary Committee, did not intend to allow the question of a pension scheme to be dropped.

### Lunacy Increasing in Bulk.

Lunacy, Dr. Newington continued, is increasing in bulk, and the British public is naturally very sensitive about the treatment of its own relatives, for this is just where a more personal feeling comes in. There is hardly a village in which someone has not a relative in an asylum, and as the efficiency of the care bestowed upon them is dependent upon the status of the nursing staff, the importance of the matter of superannuation is at once evident. To quote from the annual report: "The introduction in the session of 1900 of a Lunacy Bill by the Lord Chancellor without any clause relating to pensions for asylum workers, has necessitated representations on behalf of this association as to the desirability, on public grounds, of a system of satisfactory and assured superannuation allowances for those who have spent the best years of their life in the wearing work of tending and nursing the insane in our county and borough asylums. It is to be hoped that the House of Commons will see the wisdom of providing for the efficiency, permanence, and contentment of the public asylum service of the country by the introduction of an adequate pension clause."

### "Not as Rich as a City Company, but Still Able to Pay its Way."

This is the condition, from a financial point of view, of the Association. The "Home of Rest Fund" shows a balance of £71 10s. 4d., and nine cases have received grants during the year. In expenses, as in membership, the Association increases.

The attendance, instead of being three persons, as at the first annual meeting held, was considerable, nearly all being either workers themselves or connected in some way with asylum work.

The only change made in the officers was Miss Honnor Morten's election as hon. treasurer, in place of Miss Evans, who retired.

### To Nurses.

We invite contributions from any of our readers, and shall be glad to pay for "Notes on News from the Nursing World," or for articles describing nursing experiences, or dealing with any nursing question from an original point of view. The minimum payment for contributions is 5s., but we welcome interesting contributions of a column, or a page, in length. It may be added that notices of entertainments, presentations, and deaths are not paid for, but, of course, we are always glad to receive them. All rejected manuscripts are returned in due course, and all payments for manuscripts used are made as early as possible at the beginning of each quarter.

### Nursing of Orthopædic Cases.

Clinical Lecture delivered to Nurses at the City Orthopædic Hospital, Tuesday, May 8th, 1900, By NOBLE SMITH, F.R.C.S.Ed., Surgeon to the Hospital.

I ASSUME that you all have a thorough knowledge of the general principles of nursing, and my object will be to discuss the particular points requiring the care and attention of nurses in dealing with orthopædic cases. For practical purposes we may divide the cases that come to this hospital into two classes: Firstly, those in which there is inflammation of the joints; and, secondly, those in which there is deformity without inflammation.

In the first class (where there is inflammation) the greatest care is necessary on the part of the nurse in handling the patient. She must observe the utmost gentleness, and above all things must avoid moving the inflamed joint or joints.

In the second class of cases, which embraces a very great variety of affections, this extreme care is not necessary; in fact, in a great many of them, certain well-regulated movements, exercises, and massage are very beneficial.

The division of cases under these two classes is not a usual one to make, and I have only adopted it for the purpose of emphasising the great importance which attaches to the nursing of active joint disease.

A large majority of the diseased joints with which we have to deal are those of tubercular origin, but you must not confound tuberculous disease of the bones and joints with tuberculous disease of the lungs, because bones and joints are far more easily cured than are the very delicate tissues of the lungs.

I would ask you to look upon every patient suffering from joint disease as curable. Some, indeed, will come under your care whose constitutions are so much undermined by tuberculous disease that their recovery may be hopeless or nearly so; that is a condition which we term "General Tuberculosis," but the case must be very bad indeed to be absolutely hopeless.

I will now consider tuberculous disease of the spine, commonly called "Spinal Caries," which we may perhaps view as the most important, because several joints are usually involved in the disease, and also because of the proximity to the diseased parts of that important nerve centre the spinal cord. The object of treatment is, first and foremost, rest of these diseased parts, and it is impossible to rest any two vertebræ completely without supporting the whole spine. Rest in bed alone is not sufficient, as every movement of the patient moves the spine.

### SPLINT.

The apparatus used for this purpose must extend far beyond the affected parts. You all know the way in which such treatment is carried out in this hospital, and as the adjustments of the splints are carried out by the surgeons, it does not devolve on you to alter their shape or position. It is, however, most necessary that you should understand what is intended to be effected by the support, and to realise the necessity of using the utmost care so as not to disturb the diseased parts at any time that the splint may have to be removed, or even when it is being worn.

### LIFTING AND CARRYING.

In carrying or lifting the patient the whole spine is to be evenly supported, and this can only be thoroughly accomplished by lifting the patient on the supporting apparatus as described below. The nurse must also understand that separate movements of the arms and legs will influence the spine, and that even the head must not be allowed to be unsupported when the child is horizontal. If the thighs are supported, slightly flexed upon the body, the legs may hang at a right angle to them, but the thighs must not be bent up upon the abdomen. When the patient sits up—supposing he is well enough to do so—it is important that the knees should be bent, as, if they are extended, the spine is necessarily bowed backwards. (Here Mr. Noble Smith demonstrated the positions by means of a patient.) Of course, the instrument will prevent the back bending posteriorly very much, but still the strain will be severe and will do harm. All twisting of the body is to be very carefully avoided. When recumbent, the legs and hips must not be turned laterally without the body being turned also. Great care is necessary to carry the patient safely; the arms of the nurse should support both extremities of the instrument, or the right arm may take all the instrument and the left the thighs, but it is well that the ends of the fingers of the left hand should just grasp the lower end of the splint. One arm should be placed beneath the patient's shoulders, the head resting against the upper arm, the other hand supporting the back of the pelvic belt, and this arm should support the thighs. By this means the spine may be prevented almost absolutely from movement. It is of the utmost importance that the nurse should observe the greatest care and tenderness in moving the patient. The slightest jolt should be guarded against, and every care taken to avoid producing any pain.

### WASHING.

General washing of the patient should be carried out without disturbing the spine. In referring to this subject in my book, "Spinal Caries," I have stated that if the surgeon is not sure about the carefulness of the nurse, it will be well that the washing be considerably limited rather than the patient be subjected to the risk of injury of the diseased part. Powdering the body thoroughly with prepared Fuller's earth will take the place of washing to a great extent, but still a certain amount of ablution is absolutely necessary. To wash the patient, we will assume that he is in the prone position, lying thoroughly supported on his couch or mattress, his arms resting on a pillow, and his head on his arms. Then the instrument is to be unbuckled. The straps of both arms, the straps on one side of the abdomen, and the strap of the pelvic belt being unfastened, the instrument can be lifted sideways as if it were a lid, but not in any other way removed. The back of the patient is then to be washed carefully, dried, and the instrument buckled on again. Every buckle having been carefully fastened, the patient is then to be lifted up, and placed on his back on a flat bed or mattress, taking care that he is comfortably supported. Then the straps should be again unbuckled, and the front of the body exposed and washed, and the straps again fastened.

### ATTENTION TO FUNCTIONS.

Defæcation and micturition are to be carefully attended to, and the nurse should manage without disturbing the patient unduly. It is not difficult for these matters to be attended to on the prone couch, but it is sometimes preferred to attend to the bowels while the patient is resting on his back in bed. For that purpose an ordinary small bedpan is available, and it is a good plan to let the patient lie on two mattresses separated slightly in such a manner that the bedpan can be passed between the two, so that the patient need not be raised up while it is being used. The two most important points in the treatment of caries of the spine are: 1st. Rest of the diseased parts; 2nd. Restoration and maintenance of the patient's vitality. You have heard a great deal about the open-air treatment of tuberculosis, but, in respect to tuberculous disease of bones and joints, we have ample evidence that cases can get perfectly well, even in the worst parts of London, if the patient's vitality be kept up, and the diseased parts be efficiently supported.

In treating tuberculous disease, it is a fight between the vitality of the patient and the disease. Tubercle bacilli are almost everywhere about us, and attack those whose vitality is low. Food of the most nourishing quality is to be persevered with, and the patient's strength increased in every possible way.

### Echoes from the Outside World.

### AN OPEN LETTER TO A HOSPITAL NURSE.

It is said that it is always the unexpected which happens, and lately the truth of the saying has been demonstrated once more. For some time every effort appears to have been made by the Boers to strengthen the fortifications around Kroonstad, so successfully that the position was described as "practically impregnable," and we have looked forward with a certain amount of dread to the battle which all the papers declared to be imminent. Then, quite suddenly, came the news that the far-extending sweep of Lord Roberts's flanking operations had been too much for the enemy, and they had fled, leaving our army to occupy the town without resistance. Surrounded by his body guard of Colonials, and followed by the Guards and the 18th Brigade, the Commander-in-Chief made a triumphal entry, welcomed by the few British residents in the town, and now the British flag flies over what was the Free State capital for the second time. Ex-President Steyn appears to have done his best—or his worst—to make his burghers resist. He threatened and he cajoled, and finally, losing his temper, he took to kicking and cuffing them. But still they refused to fight, preferring to run; so he ran too, and has now proclaimed that the seat of the Free State Government has been moved again, on this occasion to Heilbron. Where next? Since then we have had the news of brilliant achievements in Natal by General Buller, who has reoccupied Dundee and Glencoe, and who, by these masterly strokes, has silenced his critics.

I wish I were a personal friend of General Gatacre. Then I should have had some excuse for going to meet and welcome him at Waterloo on Saturday; but, being a total stranger, it would, I felt, have been nothing but presumption to try and show my sympathy, and so I stayed away. But I am so sorry for him. Supposing that he has made mistakes, so have other people, and a man can only do his best. To be singled out as the one scapegoat of the army is awfully hard lines, and it is pleasant to see him spoken of in a private letter from the front—which must have come by the same mail as himself—as "a gallant officer and a perfect gentleman." To turn to a brighter subject, do you see that two subscription lists have been started for the hero of Mafeking? One, which is unlimited as to amount, has been opened by Lady Georgiana Curzon—sister of Lady Sarah Wilson, who has been inside the besieged city for months—in order that a large sum may be placed in the hands of Colonel Baden Powell, directly he is free, to spend in the alleviation of distress amongst the inhabitants of the town. The other is restricted to shillings, which may be given only by unmarried ladies, and expended in a sword of honour for "the dear man," as the proposer of the scheme, a girl of 18, calls "B. P." I know to which most of you would prefer to send your mite.

The successful scholars of the Girls' Public Day School Company were in luck's way last week. Princess Louise had promised some time back to distribute to them their prizes. Then came the death of the Duke of Argyll, and the consequent cancelling of all his daughter-in-law's engagements. The spirits of those who had counted upon receiving their gifts from Royal hands went down to the lowest ebb, when suddenly a better fate than they had dared to dream of befell them. The Princess of Wales consented to take her kinswoman's place, as she had done upon a previous occasion in 1883, and on Thursday, looking very sweet and altogether charming in a little violet toque and a most becoming violet boa, "Our Princess," with her husband and daughter, appeared at the Albert Hall. She soon found that it was no light task she had undertaken, so large was the army of triumphant scholars, but to judge by the countenances of the girls as they went back to their places, she must have given away a great deal of pleasure as well as a goodly number of books. I could not help comparing the countenances of the going and returning pupils. The ordeal of having to bow to a Princess was evidently to many very formidable, but when the fearful yet joyful moment was past, radiant happiness illuminated every face. One man amongst several women is generally an object of compassion, but the one boy who came at the tail of the 3,500 girls seemed on this occasion to be principally an object of amusement. His dignity was so delightful, he was not the slightest bit flurried, notwithstanding—or perhaps in spite of—his tender years, and evidently the Princess was much entertained with the demeanour of the dear little man, for she laughed as he came towards her and patted him on the back as he left.

All of us who can will probably go to the Woman's Exhibition, because we naturally like to know all that there is to know about ourselves. The question seems to be, rather, whether the new show at Earl's Court will be a success from a male point of view. It may have been because novelty is sometimes unacceptable simply on account of its newness, but when I was there the other day I heard some very decided opinions on the part of the young men around me. There were several things the majority did not like, and I must say I felt a little pleased to hear that they objected to women having to run beside the little ponies when tired and breathless; to women acting as wine stewards; and to women, attired in stiff shirt fronts, white ties, and neat little jackets—genuine male waiters except for their skirts. As gendarmes—or do they fancy they act the part of the humble policeman?—of course they are a dead failure. For a guardian of the peace, finding her authoritative "Pass on" is not attended to, to be obliged to resort to persuasion and intensely female remonstrance is ridiculous, and the sooner a few of the sterner sex act as "movers-on" the better. Also there seemed to be a good deal of grumbling at the bands. The war has made everyone so warlike that no musicians, however well they play, can take the place of a military band with men in military costume, and the management, if it be wise, will leaven the lump of femininity here and there with a little of the masculine element.

As a show, there is much to interest already, and a great deal more yet to come. There are pictures of women and pictures by women; there is tapestry and silk embroidery; metal work and carving—all, of course, done by women. There is a collection of dolls which will delight every child, and most women in the kingdom—a whole theatre full. The Queen of Roumania has forwarded her enormous family, some dressed by herself and some dressed by nearly every crowned head in Europe, including our own Queen herself. Then there is the living show of the "Women of All Nations," each group being placed amid appropriate scenery and surroundings. England is represented by a drawing-room where ladies sit on spindle-legged chairs and do crochet work (I had hoped that English women had progressed a little beyond that sort of thing, but such is life *à l'Anglaise* according to Mr. Imre Kiralfy). German ladies are drinking coffee and knitting, Spanish dames fanning themselves in yellow petticoats, Swiss girls are climbing mountains in high-heeled (!) shoes, whilst the women of the Eastern countries are doing little but look nice. Especially interesting to nurses is the hospital department, with its field hospital fully equipped for service on the battle-field.

### A Book and its Story.

### MISS ELLEN THORNEYCROFT-FOWLER'S NEW NOVEL.

It is in the heart of Mershire, a district of the Midland Black Country well known to the authoress, that Miss Fowler has laid the scene of her new novel.* The flame and cloud of the chimneys and furnaces of Osierfield form a lurid background to the peaceful, pastoral country lying in the near distance like a glad ray of light in the foreground of a scene which would otherwise be barren enough. No change of the seasons is visible on the smoke-grimed, desolate trees; everywhere there are signs of the sinister effects on nature of the murky, suffocating smoke, which issues with ceaseless energy from tall chimneys overlooking the landscape. Under the shadow of this clouded district lived Elisabeth Farringdon with her maiden cousins Anne and Maria, daughters and co-heiresses of John Farringdon, late owner of the Osierfield Ironworks, which, in the good old days when Mershire made iron for half the world, were the largest works in the county. The Farringdons had owned these works for several generations, and were, in consequence, looked upon as the royal family of Sedghill. Miss Fowler here, as in previous books, introduces her readers to the inner circle of Methodism. The Farringdons were Methodists of the stern, unprogressive type which was peculiarly trying to the restless, active-brained little Elisabeth, whose insatiable craving for "finding out" things was stifled by the narrow groove in which her relatives moved. The soil of Nonconformity is not one in which the characteristics of a complex, artistic temperament can develop fully.

Elisabeth's unregenerate heart was stirred early by the desire for personal beauty. On their way to chapel one Sunday she opens her heart to Miss Farringdon. "Oh! Cousin Maria, I do wish I was pretty." Elisabeth was "never afraid of anybody," otherwise she would have paused before expressing such a frivolous desire before her cousin. And "it was the absence of fear in Elisabeth which made her so dear to the heart of the severe Miss Farringdon." "That is a vain wish, my child. Favour is deceitful, and beauty is vain, and the Lord looketh on the heart and not on the outward countenance." Elisabeth replied briefly, "I was not thinking of the Lord. I was thinking of other people; and they love you so much more if you are pretty than if you are not." "That is not so," said Miss Farringdon, and she believed she was speaking the truth.

Elisabeth's life had compensations in "a comfortable friendship" for Christopher Thornley, whose uncle was her cousin's, Miss Farringdon's, business manager.

"Unlike Elisabeth, Christopher had a passion for righteousness and for honour, but no power of artistic perception. His standard was whether things were right or wrong, honourable or dishonourable; hers was whether they were beautiful or ugly, pleasant or unpleasant. Consequently the two moved along parallel lines, and she moved a good deal more quickly than he did. Christopher had deep convictions, but was very shy of expressing them. Elisabeth's convictions were not particularly deep, but, such as they were, all the world was welcome to them so far as she was concerned." The contrast comes out further. "He was always trying to do right, and she only when she thought about it. Nevertheless, when she did give her attention to the matter, she had much more beautiful and comforting thoughts than he had."

Elisabeth and Christopher were invited to a tea-party at the house of some humble members of the connexion to which they belong. If we had not met with similar scenes and characters, the description of the hospitable Mrs. Bateson and her guests would be more amusing than it is. The most impartial critic becomes weary of conversations in which petty domestic details, mingled with illustrative texts, applied indiscriminately, are delivered with sanctimoniousness and an asperity peculiar to the class. But Elisabeth was before all things a sociable being, and revelled in the small festivities which came in her way. "Mrs. Bateson had killed a pig, and pork pies were for tea." The brunt of the conversation on this occasion was borne by the two worthy ladies, and Elisabeth had only casual a opportunity of breaking in; but this opportunity she never missed. Her comments on the description of a recent wedding of a widower, given by Mrs. Hankey, are worthy of record:—

"The wedding was beautiful; I never saw its equal—never; and as for the prayer the minister offered up at the end of the service—well, such a lot of information about love and marriage; the like of it I never heard before. And when he drew a picture of the bridegroom's first wife, and how she would be waiting to welcome them both on the further shore, upon my word there wasn't a dry eye in the chapel." Elisabeth inquired speculatively, "What did Susan say? I expect she didn't want another wife to welcome them on the further shore?" Mrs. Hankey, in reply, reads her a little homily on the sordid condition of a mind which could conceive the idea of wanting, even in heaven, to keep everything to itself and not sharing it with others. On matrimony, and men in general, Elisabeth heard for the first time some clear and distinct views:—

"Well, I holds to folks getting married; it gives 'em something to think about, and if folks has nothing to think about they think about mischief." "That's true, especially if they happen to be men." "Why do men think about mischief more than women?" asked Elisabeth. "Because the Lord made 'em so, and it's not for us to complain," said Mrs. Bateson, which was a resigned way of bending to the inevitable.

As Elisabeth develops her artistic leanings become pronounced. She has one desire, that of becoming a distinguished artist, to tell to others through her pictures something of the beauty, the pathos, and the tragedy of life, which she felt so keenly. Her strong nature, sensitive and imaginative as it was, looked at existence and all that goes up to make it merely as necessary adjuncts to the perfection of her artistic creations. Love even was to be subservient to this end. "I mean to fall in love because everyone else does, but I shall do it just as an experience to make me paint better pictures. . . . But it won't be as important to me as my art. . . . It must be far lovelier to tell all your thoughts to the whole world, and know that the whole world will understand and be interested in them." Of course, lovers come into her life, but "though several men had wanted to marry Elisabeth few had succeeded in winning her." She had her art to fall back upon, and succeeded in becoming famous through it. Upon the death of her cousins she holds their property in trust until a missing heir is found. Christopher goes in search of him, but he is found nearer home in an unexpected person, whom Elizabeth wins back from the shadows of death by her love. As a study, her character, next to Christopher's, is the most interesting in the book. Alan Tremaine, the socialist, and Cecil Farquahar, the artist, are also consistent delineations. "The Farringdons" will not add to Miss Fowler's reputation, nor will it diminish from it. Had it run in less familiar grooves it would have been more effective. But to those readers who may not have seen her previous novels the present one will prove readable enough.

* "The Farringdons." By E. Thorneycroft-Fowler. One vol. Price 6s. (Hutchinson and Co.)

### Everybody's Opinion.

[Correspondence on all subjects is invited, but we cannot in any way be responsible for the opinions expressed by our correspondents. No communication can be entertained if the name and address of the correspondent is not given, as a guarantee of good faith but not necessarily for publication, or unless one side of the paper only is written on.]

### CANON GORE AND NURSES.

"L. C." WRITES: While Canon Gore was in residence at Westminster Abbey, I was able, through the kindness of my matron, to secure several Sundays off, and was always able to get a good seat. But some of my friends could not get off duty in time to go early enough to obtain a good seat within hearing distance of the pulpit. I wanted them to go, so I wrote to the Canon and told him my difficulty. By return of post he sent a pass for two. Twice he did this, and his kindness was greatly appreciated.

### THE ROYAL NATIONAL PENSION FUND.

MISS GARRETT, lady superintendent of the Ipswich Nurses' Home, writes that she wishes through the pages of THE HOSPITAL to heartily thank the managers of the Royal National Pension Fund for the kindness and courtesy two of her nurses have lately received. She continues: "Unfortunately these two nurses had only recently joined for sick pay when they contracted long illnesses. As soon as their names went to the office the weekly payments were sent with a letter of sympathy, and each week the money came without any trouble. Trained nurses have every reason to regard Sir Henry Burdett with the greatest gratitude as a friend in need for having founded and so successfully carried on such a boon to nurses as the Pension Fund." To show what can be done by thrift Miss Garrett adds that with one exception all her nurses who have joined the Fund are those at present earning the smallest salaries.

### "MORE AMATEUR INTERFERENCE."

MISS S. BUCHANAN-RIDDELL writes from 9, Sloane Gardens, S.W.: Would you kindly allow me a corner in your valuable paper? Somebody has sent me a cutting from last week's HOSPITAL, dated May 12th, headed "More Amateur Interference," and calling my little appeal "a remarkable intimation." I am a bad grammarian at the best, but I really do not quite see why the kind editor of the *Times* should not publish any number of intimations if it pleases him to do so, and in this case I am particularly grateful for his kindness, as it has enabled me to pay the second-class fares of Sisters Stella and Martha, who were cabled for by my sister—Sister May—to come out at once to her at the Camp Hospital, Ladysmith, owing to the death of Sister Theresa, who, after working twelve hours a day in the Base Hospital, Maritzburg, went with the other sisters to Intombi Camp, and there contracted dysentery from the infectious state of the camp, and also from the awful stench which seemed to affect her from the first. She was taken back to Maritzburg, and nursed by some French sisters in a lovely private hospital, but on April 9th she became dangerously ill and passed to her rest, having, indeed, laid down her life for the sake of her patients. Although only a volunteer nurse she was thought highly of for her work through the long hot five months from November to April, and was given a funeral with military honours—her coffin draped in the Union Jack, carried to the grave by soldiers, and a volley fired over it. Perhaps I may here be allowed to give "an intimation" that Sister Theresa was a most capable, good nurse and woman, and I believe the London Hospital is proud to claim the privilege of having trained her, and after being a matron and holding certificates from many doctors, including Mr. Treves, she joined the Sisters of the Church, and was one of the little band of workers whom the *Daily Mail* and C.O.S. warned the public against supporting as uncertificated humbugs! Nothing daunted, they went out, and have been working ever since under military authorities. Perhaps your readers may like to hear Sister May's letter, which came the day after my appeal was published: "Tin Camp, Natal, April 14th.—We are delighted with the large case of stores and comforts which has just arrived; the traffic is still very slow from Maritzburg. The case containing mattresses, sheets, &c., are invaluable. The hospitals are three in number, known as 18, 11, and 24. There is a head doctor for each hospital, and an army surgeon, under him an Eurasian assistant, under him a native apothecary, who gives all medicines, fomentations, &c., and English orderlies complete the staff. Sister Clare and Sister Evangeline each have a ward with bad cases, and I am attached to No. 18, with a free hand to do all I can for the other eight wards. The doctors and orderlies are so kind and thoughtful, the patients are cheering up. The work at first seemed so overwhelming, with over 300. We are so thankful to provide extra comforts the men want." I think these extracts will show that I have a fair reason for asking for funds, as even fresh workers are better than none, and the sisters have to do cooking and cleaning as well as "smoothing the pillows," which, being "an amateur," is probably my idea of nursing. I do not in the least mind being ridiculed myself, but I do think it is a pity that such a valuable periodical as THE HOSPITAL should descend to jeer at even an amateur. I have many friends who are following that most noble of all professions—nursing the sick and suffering—and I think that any man or woman who is trying to make others happier should meet with courtesy and respect. Pardon my taking the liberty of writing.

*** There was, of course, no idea on our part of jeering at anyone, and we are quite sure that Miss Buchanan-Riddell, in collecting funds to send out workers and stores to assist the overstrained nurses in the Natal military hospitals, is actuated by the kindliest of motives. But we know no reason why she should despatch nurses to South Africa any more than any other private individual; nor, unless the sister-in-law of Miss Buchanan-Riddell is a trained nurse employed by the War Office authorities and deputed by them, has she any *locus standi* in a military hospital, "nursing and tending the sick and wounded."—ED. *T. H.*

### Death in Our Ranks.

WE regret to record the death of Nurse Beatrice Abraham, which took place at the Chorlton Hospital, West Didsbury, on Saturday morning the 12th instant, of acute pneumonia, after a few days' illness, at the early age of 22. Although having been at the hospital but a short time she had, says a correspondent, endeared herself to her fellow nurses, and by her exemplary conduct and loving disposition had won the respect and esteem of all with whom she came in contact. She was laid to rest in the Southern Cemetery, the first part of the service being held in the hospital chapel, where the large majority of the staff assembled to pay their last tribute of love to their fellow-worker." It is very said that a young probationer, said to be a strong, healthy girl, should have succumbed to pneumonia. Young nurses cannot be too careful to apply to their matrons on the occurrence of earliest symptoms of illness, and it is urgently important that matrons should deal carefully with even what may appear trivial complaints among their staff. Too often we fear that plucky probationers struggle on against the beginning of illness from a feeling of dislike to give in, and the slightest lack of sympathy on the part of matrons or unwillingness to let nurses lie up for small ailments may have very serious consequences.

### Where to Go.

NATIONAL BAZAAR, EMPRESS ROOMS, ROYAL PALACE HOTEL, KENSINGTON.—May 24th, half-past two p.m., opening by the Princess of Wales. May 25th, two p.m., opening by the Duke of Cambridge. May 26th, two p.m., opening by General Sir George White.

LORD LEIGHTON'S HOUSE, 2, HOLLAND PARK ROAD, KENSINGTON, W.—Tuesdays and Saturdays, free; other days, 1s. Hours, two to half-past five p.m.

NATIONAL HOME-READING UNION.—Meeting at Salters' Hall, St. Swithin's Lane, Friday, May 18, at half-past four p.m.

### For Reading to the Sick.

"ENDURANCE is the crowning quality, and patience all the passion of great hearts."—*Lowell.*

He does not fail
For thy impatience, but stands by thee still—
Patient, unfaltering, till thou shalt grow
Patient—and wouldst not miss the sharpness grown
To custom, which assures Him at thy side.
—*H. H. King.*

Lord, what am I, that with unceasing care
Thou didst seek after me—that Thou didst wait,
Wet with unhealthy dew before my gate
And pass the gloomy nights of winter there?
How oft my guardian angel gently cried—
"Soul, from thy casement look, and thou shalt see
How He persists to watch and wait for thee."
And, oh! how often to that voice of sorrow,
"To-morrow we will open," I replied?
And when the morrow came I answered still—
"To-morrow." —*Longfellow.*

From bearing light
Our sorest burdens, comes fresh strength to bear;
And so we rise again towards the light,
And quit the sunless depths for upper air.
Meek patience is as diver's breath to all
Who sink in sorrow's sea, and many a ray
Comes gleaming downward from the source of day
To guide us re-ascending from our fall. —*Turner.*

**Reading.**

We cannot always be doing a great work, but we can always be doing something that belongs to our condition. To be silent, to suffer, to pray when we cannot act, is acceptable to God. A disappointment, a contradiction, a harsh word, an annoyance, a wrong received and endured as in His presence, is worth more than a long prayer; and we do not lose time if we bear its loss with gentleness and patience, provided the loss was inevitable and not caused by our own fault.—*Fenelon.*

"There shall no evil befall thee, neither shall any plague come nigh thy dwelling," is a promise to the fullest extent verified in the case of all "who dwell in the secret place of the Most High." To them sorrows are not "evils," sicknesses are not "plagues;" the shadow of the Almighty extending far around those who abide under it alters the character of all things which come within its influence.—*Anon.*

God makes every common thing serve, if thou wilt to enlarge that capacity of bliss in His love. Not a prayer, not an act of faithfulness in your calling, not a self-denying, or kind word or deed, done out of love for Himself; not a weariness or painfulness endured patiently; not a duty performed; not a temptation resisted, but it enlarges the whole soul for the endless capacity of the love of God.—*Pusey.*

Who knows? God knows; and what He knows
Is well and best.
The darkness hideth not from Him, but glows
Clear as the morning or the evening rose
Of east or west.

Wherefore man's strength is to sit still;
Not wasting care
To antidote to-morrow's good or ill,
Yet watching meekly with good will,
Watching to prayer.

Some rising or some setting ray
From east to west,
If not to-day, why, then, another day,
Will light each dove upon the homeward way
Safe to her nest. *C. Rossetti.*

### Notes and Queries.

THE Editor is always willing to answer in this column, without any fee, all reasonable questions, as soon as possible.

But the following rules must be carefully observed:—

1. Every communication must be accompanied by the name and address of the writer.
2. The question must always bear upon nursing, directly or indirectly.

If an answer is required by letter a fee of half-a-crown must be enclosed with the note containing the inquiry.

*Paralysed.*

(64) 1. Can you kindly recommend a home for a man suffering from paralysis? His family is very poor—the wife maintains it by laundry work—but a small annuity might be provided by the sick fund of the church and by private subscriptions. 2. Will you kindly say if nurses who have been trained in a union infirmary are accepted by the Royal National Pension Fund for Nurses, and what is the premium to be paid if a nurse is over thirty years of age? The article in this week's HOSPITAL proves that it is a satisfactory investment, if I may so term it, and I am anxious to know if all nurses can be enrolled, or is it only for nurses trained in a general infirmary?—*District Nurse.*

This seems to be a case for the local workhouse infirmary. But you can do no harm by applying to the National Hospital for the Paralysed and Epileptic, Queen's Square, Bloomsbury; and to the Hospital for Epilepsy and Paralysis, 32, Portland Terrace, Regent's Terrace, N.W. 2. All women who earn their living by nursing are eligible for the Royal National Pension Fund for Nurses. The Secretary, 28, Finsbury Pavement, E.C., will be happy to give you full particulars.

*Registered.*

(65) Will you please tell me how I can be "registered," and where?—*Nurse H.*

Apply to the Secretary, Royal British Nurses' Association, 17, Old Cavendish Street, W.

*Probationer.*

(66) Can you send me information as to which is the proper way for me to get into a hospital to learn nursing? I am not well off. I am 25 years of age, and strong.—*Emily H.*

Apply to the Matrons of the different nursing schools. If you get a copy of the "Nursing Profession; How and Where to Train," or saw one at the office of THE HOSPITAL, you would be able to select the schools most likely to suit you.

(67) 1. I am desirous of becoming a nurse, but am sorry to say I have lost my sense of smell, and wish to know if it would prevent me becoming one. 2. I should also like to know if 4ft. 10½in. would be too short.—*An Anxious One.*

1. The medical officer of the institution proposing to train you would inform you if the defect is insuperable. 2. Your height is a drawback.

*Testing.*

(68) 1. How is urine tested for blood? 2. For pus?—*E. M. S.*

1. Add a drop of tincture of guaiacum, and then a little ozonic ether to some urine in a test-tube, when, if blood be present, a blue colour will appear. 2. Add a small portion of liquor potassæ to the sediment, which will turn thick and ropy if it contains pus.

*Management of Baby.*

(69) Can any of your readers recommend me a book on the management of wet nurses; and on a baby from birth. I would like the book to combine the two.—*K. F. F.*

Braidwood's "Mother's Help and Guide" might give you the information you want.

*Training.*

(70) Can you tell me which hospital of the three mentioned is the best to train at? The Royal South Hants Infirmary, Southampton; the General Infirmary, Salisbury; or the Royal Portsmouth Hospital?—*I. H. R.*

The first-named is the only one that offers three years' training.

*Recognised Training School.*

(71) What constitutes a recognised training school?—*Exile.*

A training school to be recognised by the Local Government Board as such must maintain a resident medical officer. If in any doubt as to the status of a school, you could write direct to the Secretary of the Local Government Board, Whitehall, S.W.

*Dispensing.*

(72) Will you kindly tell me the best way and where to study to become a qualified dispenser?—*E. M. J.*

Write for advice to the Secretary, the Pharmaceutical Society, 17, Bloomsbury Square, W.C.

*Guy's School, Hertford.*

(73) Kindly tell me how to proceed to get two little girls into the above school?—*Ability.*

Write direct to the Secretary for information, stating full particulars.

**Standard Books of Reference.**

"The Nursing Profession: How and Where to Train." 2s. net.
"The Nurses' Dictionary of Medical Terms." 2s.
"Burdett's Series of Nursing Text-Books." 1s. each.
"A Handbook for Nurses." (Illustrated.) 5s.
"Nursing: Its Theory and Practice." New Edition. 3s. 6d.
"Helps in Sickness and to Health." Fifteenth Thousand. 5s.

All these are published by THE SCIENTIFIC PRESS, Ltd., and may be obtained through any bookseller or direct from the publishers, 28 & 29, Southampton Street, London, W.C.

